# The effect of underlying diseases on pneumonia risk in patients with neurogenic or tumor-related dysphagia: a retrospective cohort study

**DOI:** 10.1007/s00405-024-08815-6

**Published:** 2024-07-11

**Authors:** Almut C. Niessen, Jana Zang, Ferkhunda Tinat, Julie C. Nienstedt, Frank Müller, Till Flügel, Julia Glinzer, Christina Pflug

**Affiliations:** Universitätsklinikum Hamburg-Eppendorf, Martinistr 48-52, Hamburg, 20246 Germany

**Keywords:** Dysphagia in head and neck cancer, Neurogenic dysphagia, Pneumonia risk, Food adaptation

## Abstract

**Objective:**

To analyze the association of neurological disorders (ND) and head and neck cancer (HNC) with dysphagia severity and aspiration pneumonia occurrence.

**Method:**

Retrospective cohort study conducted at a university dysphagia center) for two consecutive years. Patients with ND or HNC were included if they had undergone a flexible endoscopic swallowing evaluation (FEES) at the dysphagia center, and at least one food consistency had been sampled and recorded. Outcomes of interest were swallowing safety, highest penetration-aspiration-score (PAS_max_), way of food intake, presence of a tracheal tube, and occurrence of pneumonia within the past two years.

**Results:**

Of 257 consecutive patients, 199 were enrolled in the study and classified according to their underlying diagnosis into ND (120 patients) or HNC (79 patients). Forty-three HNC patients (54.4%) and 54 ND patients (45%) showed critical dysphagia in FEES (PAS ≥ 6). Binary logistic regression comparing both groups showed patients with ND to be 2.31 times more likely to develop pneumonia. However, if the 32 stroke patients were excluded from the calculation, PAS_max_ remains the only significant variable affecting pneumonia risk in both groups. Liquids were the main challenge for ND patients, while aspirating HNC patients struggled with all consistencies.

**Conclusions:**

The study shows that patients with HNC and ND differ in pneumonia risk only if stroke patients are included in the ND group. If they are excluded, the PAS score is the only remaining risk factor for pneumonia. Thickening liquids may not be suitable for all dysphagic patients; individually tailored measures might be more helpful, especially for HNC patients.

## Introduction

Dysphagia is highly relevant in medical research. With an estimated prevalence of more than 590 million people worldwide [1], dysphagia considerably burdens affected patients with a reduced quality of life (QOL). Moreover, it poses an increasing financial problem for the medical care system and society [2]. Dysphagia prolongs hospital stays and increases the readmission rates of affected patients [2].

Swallowing disorders can lead to grave consequences such as malnutrition, dehydration, inadequate medication efficacy, weight loss, reduced quality of life, and aspiration pneumonia [3]. In many underlying diseases, aspiration pneumonia is the most common cause of death in affected patients, accounting for 2.3% of all deaths in the US [4, 5]. The risk of developing fulminant pneumonia due to untreated dysphagia is increased by many affected individuals being unaware of their swallowing impairment and late diagnosis of the underlying disease [6].

Considering the entire population of patients with dysphagia, two of the most frequent etiologies are neurological disorders (ND) and head and neck cancers (HNC) [7, 8]. Most of these patients suffer from persistent or progressive dysphagia, also reflected in the patient population frequenting our university dysphagia center.

Dysphagia is very frequent in HNC patients. The prevalence of dysphagia in HNC varies greatly and depends on tumor location [9], tumor size, treatment regimen (surgery, chemotherapy, radiotherapy), and assessment methods. Reported rates range between 12% and 85%, depending on tumor location [10–14].

The severity of dysphagia largely depends on tumor-associated structures [15], treatment regimen [16], and patient-specific characteristics such as age. Another factor is the time that has elapsed since tumor therapy [13, 17], which influences whether there have been improvements in swallowing ability [18], stabilization, or even - due to side effects after irradiation or decreasing laryngeal edema - a deterioration of the swallowing function [19, 20].

Despite the decreasing incidence of laryngeal carcinoma, oropharyngeal carcinoma (OPC) incidence is increasing due to the growing group of HPV-positive OPC patients [9]. This group differs from HPV-negative patients with tobacco- and alcohol-related carcinomas in that they are younger, suffer fewer comorbidities, and have a better prognosis [21]. Survivors may, therefore, live for a long time with a significant burden of dysphagia with severely impaired quality of life, which may increase in severity over time [22]. Thus, the prevalence of dysphagia in patients with HNC is expected to increase [9].

In contrast to HNC patients, who usually suffer from chronic dysphagia that is rarely progressive, the course and occurrence of dysphagia in ND patients are very different depending on the underlying disease. Post-stroke patients, for example, may suffer from acute dysphagia with sometimes acutely life-threatening aspiration. Complete recovery from dysphagia is possible, as is the development of chronic dysphagia, which may even worsen with age [23]. Prevalence for dysphagia in patients with stroke ranges between 45% and 78% [24]. In contrast, patients with Parkinson’s disease (PD) show abnormalities in swallowing early in the course of the disease [25, 26], but severe dysphagia tends to occur mainly in later stages [27]. Prevalence rates up to 87% are reported [26, 28]. Patients with an unfavorable prognosis, such as in amyotrophic lateral sclerosis (ALS) [29], are usually dysphagic later in the course of the disease. However, this period is generally shorter compared to other chronic illnesses. The severity of dysphagia in patients with myasthenia gravis often depends on the adequacy of treatment [30].

Moreover, ND patients with diseases of different etiology are frequently unaware of existing swallowing impairment, often manifesting as severe dysphagia with silent aspiration [26].

Aging and frailty also significantly negatively impact both patient groups [31, 32], especially when combined with reduced cognitive abilities [33, 34].

The age-adjusted mortality rate for aspiration pneumonia in the US is 21.9 per 100,000 population, increasing to 643.84 per 100,000 in patients over 85 years. In 6.1% of cases where aspiration pneumonia is listed only as a contributing factor, neoplasms were the underlying cause of death [4]. If aspiration pneumonia is the primary cause of death, the same publication lists cerebrovascular disease in 11.7% and PD in 4.7% of all aspiration pneumonia-related deaths in the US. In comparison, only 1.5% of these cases suffer from HNC.

To date, it is still being determined which patients develop aspiration pneumonia and when. It might be assumed that patients who show (silent) aspiration are more likely to develop pneumonia in the course of their disease. Clinical observation suggests that HNC patients are less likely to develop pneumonia than ND patients, although they tend to show severe dysphagia with aspiration more often. The influence of the underlying disease on aspiration risk in dysphagic patients has not been studied yet. Hence, this study aimed to analyze the association of HNC and ND diagnoses with dysphagia severity and aspiration pneumonia occurrence. The data should allow conclusions for clinical practice, therapy, and assessment of pneumonia risk.

## Materials and methods

### Study design and setting

This retrospective cohort study, conducted over 15 months at a university dysphagia center, is reported following the STROBE statement [35]. It was conducted on previously anonymized data and in accordance with the Helsinki Declaration.

### Eligibility

Patients with either ND or HNC were eligible for inclusion if they had undergone a flexible endoscopic evaluation of swallowing (FEES) [36] at the dysphagia center and at least one food consistency had been tested and recorded. The exclusion criteria were the coincidence of both HNC and ND, as well as dysphagia of other etiology. No age restrictions were set.

### Variables

The outcomes of interest were swallowing safety, way of food intake, existence of a tracheal tube, and occurrence of diagnostically proven pneumonia within the previous two years.

### Data sources and measurement

One author (FT) extracted data from digital patient records and immediately anonymized them for analysis. The first assessment that fit the inclusion criteria was used in cases with multiple assessments during the analyzed time span.

### Quantitative variables

The following quantitative variables were extracted from the medical records and anonymized: age, gender, pneumonia within the last two years, presence of a feeding tube (nasogastric or percutaneous endoscopic gastrostomy (PEG)), way of food intake (oral, partially oral, non-oral), presence of a tracheal tube, and Penetration-Aspiration Scale (PAS) scores [37] for every tested consistency. Only patients with partial oral or non-oral food intake were provided with a feeding tube. All patients with a nasogastric tube were fed completely nil by mouth. Diagnosis as a qualitative variable was translated into a quantitative value by classification into the two main groups.

In cases of missing PAS scores, the recording was reassessed by the medical doctor (MD) who had performed the examination. All MDs had several years of endoscopy experience. FEES assessments are routinely compared in the department to ensure their consistent quality. For all included patients, at least one FEES recording was available.

PAS scores resulted from the following FEES protocol: Nasal decongestant and local anesthesia were initially applied in the naris examined. In the subsequent endoscopy, different colored consistencies were given in the following sequence and, if necessary, adapted to the individual needs of the patient: (i) 5 ml of semiliquid puree (IDDSI 4), (ii) 5 ml of water, (iii) 90 ml of water from a cup with a straw, (iv) half a slice of bread spread with butter or one cookie (IDDSI 7). If the patient consented, the relevant consistency was repeated in case of a penetration or aspiration.

The endoscope used was the ENF-V3 CCD rhino-laryngo-videoscope with a distal diameter of 2.6 mm (ENF-V3, Olympus Medical Systems Corp., Tokyo, Japan). For offline image analyses, FEES recordings were stored on rpSzene System v.6 (Rehder, Hamburg, Germany).

Swallowing safety was evaluated for each consistency in each patient using the PAS. The highest PAS score for each consistency and patient was selected and classified according to severity. PAS 1–2 was graded as inconspicuous, 3–5 as penetration, and 6–8 as aspiration. PAS ≥ 3 was defined as pathological, and PAS ≥ 6 was classified as critical dysphagia.

### Bias

A bias in favor of dysphagia cannot be excluded due to the study design, as patients were explicitly sent to the dysphagia center to assess suspected dysphagia, subjective signs of dysphagia, or in connection with pneumonia.

### Statistical methods

Statistical analysis was performed using SPSS Statistics version 27 (IBM, USA). Descriptive analysis was conducted for the number of cases, the sample profile (age, gender, underlying disease), functional swallowing pathologies (aspiration, penetration), and the resulting individual swallowing impairment. For comparison of dysphagia severity between the HNC and ND groups, the highest PAS value of the three tested consistencies - fluid (fld), semiliquid puree (slq), solid (sol) - was determined for each patient (PAS_max_). For further analyses, PAS values of individual consistencies were considered to compare swallowing safety for different consistencies between groups.

Subsequently, ND subgroups were analyzed for differences due to different neurological diagnoses. The stroke subgroup was excluded to control for the influence of differing disease-specific characteristics in stroke patients compared to other ND patients (age, pneumonia incidence) on the group comparison for the outcome *pneumonia incidence*.

The Mann-Whitney U test was used to test for group differences in non-parametric data. Sample sizes vary for the different independent variables and analyses due to different food consistencies not being tested in several patients. An alpha level of 0.05 was set for all statistical tests. All tests were two-tailed.

Binary logistic regression (method = enter) was calculated to test the effect of multiple independent variables on the dichotomous dependent variable (pneumonia/no pneumonia). As stroke patients show disease-specific characteristics compared with other ND patients, the subgroup of stroke patients was excluded in a second binary logistic regression.

## Results

### Patients

**Fig. 1:**
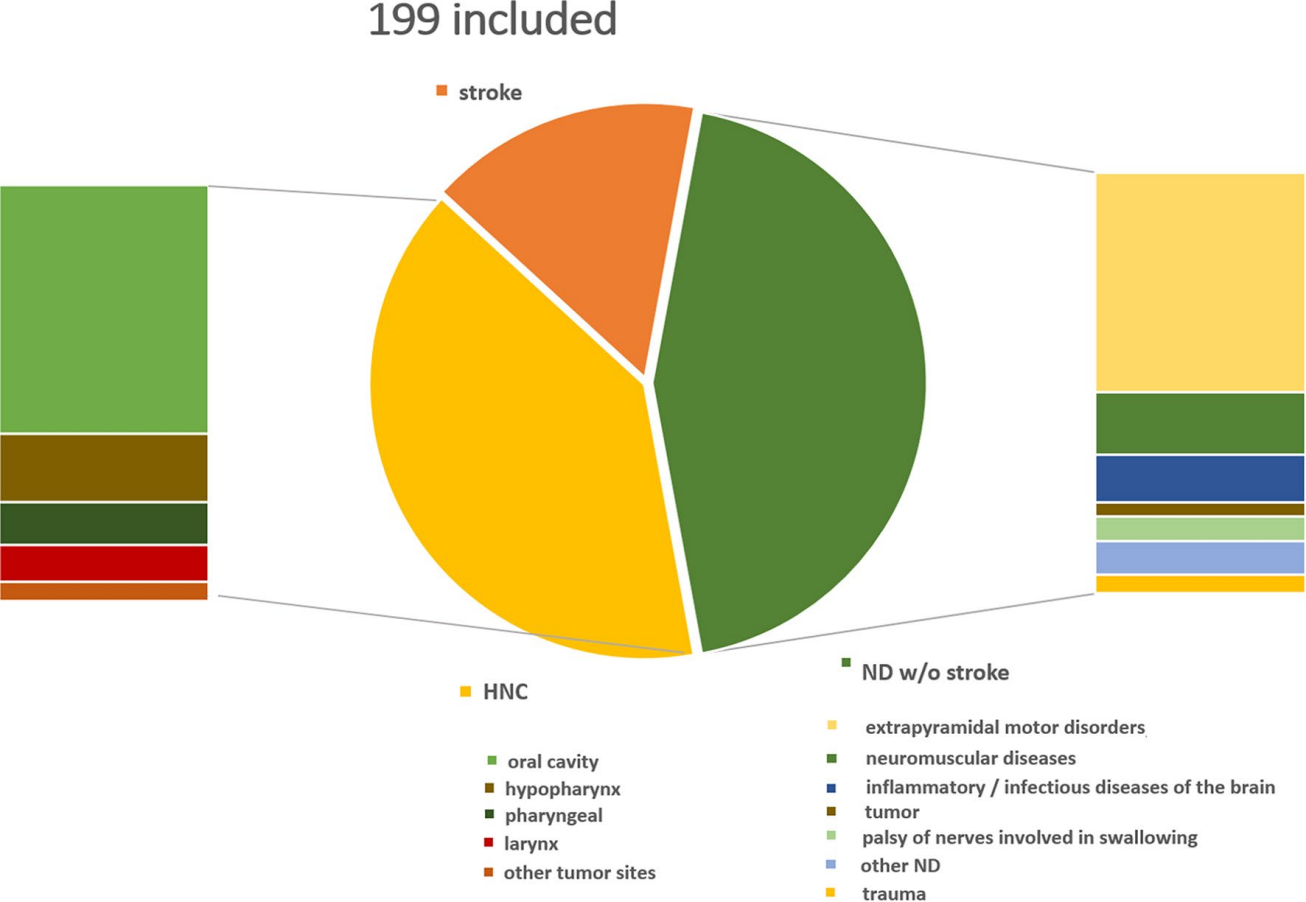
Composition of the study group. 199 Patients included in the study: Tumors in the HNC group (*n* = 79): Oral cavity (*n* = 47), hypopharynx (*n* = 13), oro- and nasopharynx (*n* = 8), larynx (*n* = 7), other (*n* = 4). Disease entities in the ND group (*n* = 120): stroke (*n* = 32), extrapyramidal motor disorders (*n* = 46), neuromuscular diseases (*n* = 13), inflammatory/infectious diseases of the brain (*n* = 10), tumor (*n* = 3), palsy of nerves involved in swallowing (*n* = 5), trauma (*n* = 4), other (*n* = 7)

Two hundred fifty-seven patients presented to our University Dysphagia Center to evaluate suspected dysphagia during the examination period. A total of 58 participants were excluded from the study, as they did not suffer from either HNC or ND (*n* = 50) or had both conditions concurrently (*n* = 8). The remaining 199 patients were grouped according to their underlying diagnosis. The ND group consisted of 120 and the HNC group of 79 patients. Most HNC patients (*n* = 70, 88.6%) had been treated with adjuvant or primary radio (-chemo)-therapy. For the detailed composition of the study group see Fig. 1.

### Sample characteristics

The main patient characteristics are presented in Table 1. In both groups, the male gender significantly predominated, accounting for at least two-thirds of patients. The median age was comparable in both groups. The total pneumonia rate was 18.6% and higher in the ND group, with 22.5% (13.9% in HNC). However, the difference between both groups did not reach statistical significance. If the total sample was divided by previous pneumonia, there were no significant differences in age and gender.

The majority of patients were on an entirely oral diet. However, more than one-third of the HNC patients and almost one-quarter of the ND patients were dependent on tube feeding.

Significantly more patients with HNC were dependent on a tracheal tube (*n* = 14, 17.7%, *U* = 3934.0, *Z* = -3.52, *p* < .001) compared to patients with ND (*n* = 4, 3.3%).

**Table 1:** Patient characteristics

	Total *n* = 199	ND *n* = 120	HNC *n* = 79
Mean Age (years ± SD)	66.26 ± 14.1	65.9 ± 16.3	66.9 ± 9.8
Gender (n (%))	m = 139 (69.8%) f = 60 (30.2%)	m = 80 (66.7%) f = 40 (33.3%)	m = 59 (74.7%) f = 20 (25.3%)
Pneumonia (n (%))	37 (18.6%)	27 (22.5%)	10 (13.9%)
Tracheal tube (n (%))	18 (10.1%)	4 (3.3%)	14 (17.7%)
Oral food intake (n (%))	148 (74.4%)	94 (78.4%)	54 (68.4%)
Partially oral food intake (n (%))	17 (8.5%)	7 (5.8%)	10 (12.6%)
Non-oral food intake (n (%))	34 (17.1%)	19 (15.8%)	15 (19.0%)

SD = standard deviation; ND = neurological disorder; HNC = head and neck cancer

Between the main groups (HNC and ND), average age, gender, provision with a feeding tube, the occurrence of pneumonia, PAS_max_, and PAS_fld_ did not differ significantly. However, significant group differences occurred for PAS_slq_ (*U* = 2274.5, *Z* = -5.08, *p* < .001), and PAS_sol_ (*U* = 1567.0, *Z* = -3.19, *p* = .001).

Considering PAS_max_, more than half of the HNC patients (54,4%, *n* = 43) showed critical dysphagia in the FEES examination. Conversely, only one-quarter (25.3%, *n* = 20) had unremarkable PAS scores (Table 2). Aspiration occurred most frequently with the liquid consistency. Puree was aspirated by 38.6% (27/70) and solids by 27.1% (13/48) patients. Interestingly, of 43 aspirating HNC patients, only eight (18.6%) had suffered from pneumonia in the past, whereas 35 (81.4%) with FEES-proven aspiration never had pneumonia.

Compared to HNC patients, in the ND group, critical dysphagia was present in 54 patients (45%). Aspiration of liquids was the most common pathology, occurring in 44% (52/119) of the patients. Semiliquid puree and solids were rarely aspirated; therefore, water was almost exclusively responsible for PAS_max_. About one-third of the ND patients (35,8%, 43/120) showed an unremarkable PAS score (PAS 1–2). Of the 54 aspirating ND patients, 18 had acquired pneumonia in the past (33.3%).

**Table 2:** FEES results (PAS) for all three consistencies in both main groups

		n=	Normal	Penetration	Aspiration	Median (Q25/Q75) ^*^
			PAS 1–2	PAS 3–5	PAS 6–8	
HNC *n* = 79	fluid	74 (93.7%)	22 (29.7%)	16 (21.6%)	36 (48.6%)	5 (2/7)
	semiliquid	70 (88.6%)	27 (38.6%)	16 (22.9%)	27 (38.6%)	4 (1/7)
	solid	48 (60.8%)	26 (54.2%)	9 (18.8%)	13 (27.1%)	1.5 (1/6)
	max.	79 (100%)	20 (25.3%)	16 (20.3%)	43 (54.4%)	6 (2.5/7.5)
ND *n* = 120	fluid	119 (99.2%)	46 (38.7%)	21 (17.6%)	52 (43.7%)	4 (1/7)
	semiliquid	112 (93.3%)	82 (73.2%)	15 (13.4%)	15 (13.4%)	1 (1/3)
	solid	91 (75.8%)	77 (84.6%)	8 (8.8%)	6 (6.6%)	1 (1/2)
	max.	120 (100%)	43 (35.8%)	23 (19.2%)	54 (45.0%)	4 (1.75/7)

ND = neurological disorder; HNC = head and neck tumor; *Interquartile range

### Consideration of the ND subgroups

Considering the ND sample, pneumonia occurred three times more often in stroke patients than in patients with other underlying neurological diseases (14/32 (44%); 13/88 (15%) respectively). Surprisingly, three stroke patients did not show aspiration in our examination, despite a reported pneumonia history, compared to seven in all pneumonia patients.

Female ND patients were significantly younger than female HNC patients (8.8 y, *p* = .009), and variance in age was markedly higher (ND: 62.1 ± 19 y / HNC: 70.9 ± 5.5 y). The age difference between the two main groups is further enhanced by the fact that the female stroke patients were significantly younger than their male counterparts (female: 58.3 ± 24.3 y. / male: 70.9 ± 9.8 y.), whereas the other neurological subgroups showed no such age differences.

### Subgroup analysis of ND and HNC excluding stroke sample

To account for the impact of various disease-specific features in stroke patients, which may differ from those in other ND patients, such as age and pneumonia incidence, we reanalyzed the statistics by excluding stroke patients and comparing ND patients without stroke to HNC patients.

The results of the ND patients, excluding stroke, compared to HNC patients, are demonstrated in Table 3. Significant differences between groups regarding tube feeding and provision with a tracheal tube remained. This was also true for the differences in swallowing safety regarding the group-specific difficulties with consistencies. Puree and solids were aspirated significantly more often by the HNC patients.

**Table 3:** Group comparison of HNC (*n* = 79) vs. ND without stroke (*n* = 88)

	*U* ^*a*^	*Z* ^b^	*p*-Value
*Age*	*3291.0*	*− 0.59*	*0.553*
*Gender*	*3131.5*	*-1.38*	*0.166*
*Feeding tube*	*2850.0*	*-2.79*	0.005*
*Tracheostomy*	*2926.0*	*-2.8*	0.005*
*PAS*_*max*_	*3063.0*	*-1.34*	*0.179*
*PAS*_*fld*_	*2906.0*	*-1.08*	*0.280*
*PAS*_*slq*_	*1522.5*	*-5.21*	0.000*
*PAS*_*sol*_	*1130.5*	*-3.29*	0.001*
*Pneumonia*	*3402.5*	*− 0.4*	*0.693*

*n* = 167, grouping variable: diagnosis HNC (79), ND without stroke (88), a-b Man-Whitney-U with associated Z value, *p < .05

### Logistic regression

The binary logistic regression was performed to assess the effects of *gender*, *age*, *PAS*_*max*_, and *diagnosis* on the likelihood that patients develop *pneumonia*. The logistic regression model was statistically significant, χ^2^(4) = 12.90, *p* < .05. The regression model correctly classified 81.4% of cases and explained 10.2% (Nagelkerke R^2^) of the variance in pneumonia development. Patients with ND were 2.31 times more likely to develop pneumonia. Patients with a higher PAS_max_ score were 1.21 times more likely to develop pneumonia (Table 4).

**Table 4:** Logistic regression (*n* = 199)

	ß^a^(SE)	OR^b^	95% CI	*p*-Value
Diagnosis	− 0.83 (0.41)	2.31	1.03–5.23	0.041*
PAS_max_	0.19 (0.07)	1.21	1.05–1.43	0.008*
Age	0.07 (0.01)	1.00	0.98–1.03	0.621
Gender	0.30 (0.43)	0.73	0.31–1.73	0.494

*Note* Dependent variable: pneumonia; in Step 1 included Variables: gender, age, PAS_max_, diagnosis; Cox & Snell R^2^ = 0.06, Nagelkerkes R^2^ = 0.10, Model χ^2^(4) = 12.90, *p* = .012 ^a^regression coefficient, ^b^Odds Ratio. ^*^p < .05

After excluding the 32 stroke patients from the ND group, a further binary logistic regression assessed the effects of *gender*, *age*, *PAS*_*max*_, and *diagnosis* on the likelihood that patients develop *pneumonia*. The regression model correctly classified 86.2% of cases and explained 9.8% (Nagelkerke R^2^) of the variance in pneumonia development. It continues to show a significant influence of the PAS_max_ on the risk of developing pneumonia (*ß*=0.22, 95% CI 1.03 to 1.51, *p* = .020) and no longer a significant influence of the underlying disease.

## Discussion

This study aimed to analyze the impact of underlying neurogenic or tumor-related disease on the risk of aspiration pneumonia risk and the severity of dysphagia.

The results of our study demonstrate that (i) HNC patients aspirate more frequently than ND patients, but the pneumonia rate is comparable in both groups. (ii) The underlying diagnosis of ND significantly influences the chance of developing pneumonia. (iii) If stroke patients are excluded from the ND group, the only remaining risk factor for pneumonia is the PAS_max_ score, thus indicating that pathological PAS increases the risk of pneumonia in all patients studied, irrespective of underlying disease. (iv) Unlike ND patients, HNC patients aspirate all consistencies similarly frequently (including semiliquid puree and solids).

Dysphagia is generally highly frequent in patients with HNC. Our study group reflects this in the significantly higher PAS values for semiliquid and solid food. Although not significant, HNC patients are also affected more severely than ND patients when considering PAS_max_ and PAS_fld_. This distinction is essential because silent aspiration is expected to carry a high risk of developing pneumonia [4].

However, the ratio of HNC patients aspirating without a history of pneumonia was higher than that of the aspirating ND patients (64% versus 51%). Thus, although HNC patients aspirate more frequently, their pneumonia rate is lower, even if the differences are not significant when considered individually. In the past, the proportion of smokers among HNC patients was extremely high. Today, in times when HPV-positive tumors are on the rise [9], this is no longer so extreme. In older HNC patients, however, this smoking habit could increase the incidence of pneumonia [38].

When including stroke patients in the ND group, the hypothesis is confirmed that ND patients are more susceptible to pneumonia despite aspirating less than HNC patients. This difference between HNC and ND patients can no longer be seen when stroke patients are excluded. The general pneumonia rate in the subgroup of stroke patients is extraordinarily high. A systematic literature review [39] shows that stroke patients, in particular, have a high incidence of pneumonia, which was confirmed in the study presented here. Stroke patients appear to have additional risk factors apart from aspiration for pneumonia in the acute stage due to the systemic immunodepression a stroke triggers [40].

The sample mainly consisted of patients presenting with chronic dysphagia. Most HNC patients had completed treatment and were no longer in the acute phase. In addition, most stroke patients were in the chronic phase, reflected in the small number (2/32) of patients being fed via NG tube. Guidelines of the German Neurological Society (DGN) recommend an NG tube if uptake of oral intake is not to be expected within seven days and a PEG tube if enteral nutrition is anticipated for more than 28 days [41]. Most of the other underlying diseases in the ND group lead to progressive deterioration during the course of the illness. Therefore, the swallowing impairment observed in this study group is likely to remain constant or is even expected to deteriorate.

There was a significant difference in the ability to swallow test boluses of different consistencies between the two groups. The HNC patients had more difficulty than the ND patients, especially with the firmer consistencies, such as semiliquid puree and solid food. However, there was no significant difference between the two groups in the ingestion of liquids.

While ND patients seem to have trouble swallowing fluids mainly due to sensory impairment, we assume that when HNC patients have problems swallowing, these are due to transport problems, among other factors. Transport problems may indeed play a more important role than actual aspiration. If HNC patients have problems swallowing, this is often true for all consistencies. The severity of symptoms depends on the tumor size and localization as well as the therapeutic regimen or surgical approach. This combination of transport problems and aspiration affects food intake and, thus, quality of life. Treating dysphagia patients with ND usually involves avoiding liquids or thickening them, but this is not always appropriate for HNC patients and may even worsen their swallowing problems.

Our data demonstrate that pathologic PAS values increase the risk of pneumonia in all dysphagic patients; even in the logistic regression excluding stroke patients, PAS_max_ remains the only significant variable influencing pneumonia risk.

This finding aligns with our expectations and the relevant literature [42]. Therefore, assessing swallowing ability using the PAS based on FEES, as performed in this study, is an essential risk assessment tool in patients suffering from dysphagia or dysphagia-associated underlying disease to estimate the probability of life-threatening pneumonia.

Tracheal cannulae obstruct swallowing by restricting laryngeal elevation and anteflexion. In daily life, a patient with a cannula will nevertheless usually eat with the cannula in place. Due to the foreign body immediately below the larynx, laryngeal sensibility is also down-regulated. This reduced sensitivity may increase the risk of aspiration since a cough reflex only occurs when the bolus reaches a tracheal region without reduced sensitivity.

Not surprisingly, the HNC group accounted for most tracheal cannulae; these patients are often cannulated primarily due to airway restriction or airway protection from bleeding postoperatively and not because of dysphagia. Similarly, they are frequently not fed orally to protect the surgical site early on. Due to this, HNC patients often receive a PEG tube during therapy, which is supposed to be removed again after the completion of treatment. Only if swallowing problems persist is the tube left in place. HNC patients are more likely to be non-orally fed than ND patients in the presented sample; these numbers are consistent with literature data [43].

Sarcopenia may increase the risk of a loss in swallowing function and difficulties returning to an oral feeding regimen after treatment [44]. While this point is relevant in a general sense, since no BMI data was collected in this study, the number of sarcopenia diagnoses in the groups cannot be specified. It is a well-established fact that sarcopenia in HNC patients leads to swallowing problems [45]. Additionally, elderly HNC patients are more susceptible to sarcopenia, particularly as radio (-chemo)-therapy increases the risk of dysphagia even further [45].

In contrast to HNC patients, conversion to non-oral feeding in ND patients is usually indicated only if severe swallowing problems occur during the course of the disease. It is also known that the subjective perception of dysphagia is significantly reduced in PD patients [26], so these patients are likely delayed in receiving therapy.

Ultimately, the goal is to prevent aspiration pneumonia by reducing the likelihood of aspiration events. In a palliative situation, the goal is to postpone them at least as much as possible. To this end, non-oral or partial oral nutrition via a PEG is usually initiated. Optimal timing is crucial for this purpose.

The higher prevalence of pneumonia in stroke patients may potentially cause an over-generalization and, thus, more restrictive treatment for all ND patients. There may also be a tendency to provide a PEG earlier for all ND patients.

Depending on the severity of dysphagia, patients may require nutritional counseling, dietary adjustments, and swallowing therapy to maintain proper nutrition and prevent weight loss. However, understanding each patient’s risk of developing aspiration pneumonia is essential to providing suitable treatment. The objective is to achieve a balance between limitations and quality of life.

### Limitations

As mentioned above, a bias in favor of dysphagia cannot be excluded, as patients were explicitly assessed for suspected dysphagia. Due to the heterogeneity of the groups, the number of cases in each subgroup is relatively small.

According to literature, the average age of women with HNC is 65 years [14], in our sample, 70.9 years. However, our findings regarding male HNC patients are consistent with existing literature [46]. The higher mean age of female HNC patients in our sample may be attributed to a sampling effect, as the rate of pneumonia in HNC patients in this cohort is consistent with the rate reported in the literature, which ranges from 5.4 to 23% [9].

In the group with unremarkable PAS values (PAS 1–2), the number of patients with previous pneumonia was unexpectedly high (7/37), especially in stroke patients (3/14). It is debatable whether the rate of false inconspicuous PAS might be high because examinations in our clinic are usually performed in the morning for logistical reasons. Fatigue may increase during the day, making aspirations more likely later in the day - a fact our study would not detect.

## Conclusion

This study offers valuable insights into the risk of aspiration pneumonia in patients with dysphagia. Despite a higher aspiration rate, HNC patients are significantly less likely to develop pneumonia than ND patients in total. However, this is no longer true when stroke patients are excluded from the ND group. Then, the PAS score remains the only factor significantly associated with aspiration pneumonia.

In contrast to ND patients, HNC patients have difficulties with all consistencies, not only liquids. Therefore, thickening fluids is not always appropriate for all patients, but the recommendations have to be tailored to the patient’s individual needs.

These results are of socio-economic significance, as both an inappropriate treatment regime to prevent aspiration pneumonia and too early or late PEG insertion represent a risk and burden for the patient and society.

## Data Availability

The data supporting this study’s findings are not openly available due to sensitivity reasons. The data supporting the findings of this study are available on reasonable request to the corresponding author and will be stored at https://www.fdr.uni-hamburg.de/.
